# Disparities in opioid treatment access and retention among women based on pregnancy status from 2006 to 2017

**DOI:** 10.1016/j.dadr.2022.100030

**Published:** 2022-02-02

**Authors:** Tenie Khachikian, Hortensia Amaro, Erick Guerrero, Yinfei Kong, Jeanne C. Marsh

**Affiliations:** a University of Chicago, School of Social Service Administration, 969 E. 60th Street, Chicago, IL 60637, USA; b Florida International University, Herbert Wertheim College of Medicine and Robert Stempel College of Public Health and Social Work, 11200 SW 8th St, AHC4, Miami, Florida 33199, USA; c I-LEAD Institute, Research to End Healthcare Disparities Corp, 12300 Wilshire Blvd, Suite 210, Los Angeles, CA 90025 USA; d California State University, Fullerton, College of Business and Economics, 800N State College Blvd, Fullerton CA 92831 USA

**Keywords:** Pregnancy, Access, Retention, Treatment, Opioid use

## Abstract

**Background:**

The purpose of this study is to assess differences in wait time and retention in opioid use disorder (OUD) treatment among a sample of pregnant and non-pregnant women from low-income urban communities in Los Angeles, California.

**Methods:**

Data were collected in 9 waves consisting of consecutive years from 2006 to 2011, and then including 2013, 2015, and 2017. The sample consisted of 12,558 women, with 285 being pregnant and 12,273 being non-pregnant. We compared pregnant women with non-pregnant women at admission on key characteristics and relied on two multilevel negative binomial regressions analyses to examine factors related to access (days on the waiting list) and retention (days in treatment).

**Results:**

We detected disparities existed in access and retention. Pregnant women spent less time waiting to initate treatment than non-pregnant women and, once in treatment, had longer treatment episodes. Among pregnant women, clients identifying as Latina or Other waited longer to enter treatment compared to clients identifying as non-Latina White or Black. Women entering residential waited longer than those entering methadone or counseling services. Pregnant women were more likely to be in treatment longer if they had mental health issues, greater parenting responsibilities (number of children less than 18), and greater SUD severity (number of prior treatment episodes).

**Conclusions:**

Findings suggest pregnant women’s access and retention can be improved through Medicaid coverage and through the implementation of a standard of care that includes MOUD (methadone) along with ancillary health and social services.

## Introduction

Opioid overdose and OUD are at epidemic levels and present a major public health crisis in the United States (Centers for Disease Control and Prevention, 2020a). OUD continues to increase at alarming rates for vulnerable populations, including pregnant women. The use of opioids during pregnancy quadrupled from 1.5 to 6.5 per 1000 hospital births from 1999 to 2014 (Agency for Healthcare Research and Quality, 2018). Research documents the negative relation of opioid use to maternal and neonatal outcomes, e.g., early labor, stillbirth, and maternal mortality (Patrick et al., 2019). Further, pregnant women face more barriers to accessing OUD treatment including lack of child care, limited family support, co-occurring mental health conditions, and unstable housing compared with non-pregnant women (Sutter et al., 2017). Research is needed to understand factors that may enhance treatment access and retention for pregnant women with OUD (Centers for Disease Control and Prevention, 2020b; Haight et al., 2018).

The standard of care for OUD treatment for pregnant and parenting women is medication to treat opioid use disorder (MOUD), including methadone, buprenorphine and naltrexone, along with comprehensive services to address mental health, trauma, economic, and social needs (such as housing and food insecurity) (Barbosa-Leiker et al., 2020; Marsh et al., 2000, 2004; Klaman et al., 2017). Methadone is the most common medication for treating OUD in pregnant women (ACOG Committee on Health Care for Underserved Women, and American Society of Addiction Medicine, 2012; Burns et al., 2007; Harter, 2019; The American College of Obstetricians and Gynecologists, 2017). Substantial research shows that OUD treatment with methadone for pregnant women increases access to prenatal care and decreases rates of opioid use and maternal mortality (Bums et al., 2007; Harter, 2019).

Additional research indicates that when buprenorphine is prescribed during pregnancy it demonstrates similar outcomes of reduced opioid use and maternal mortality (Substance Abuse and Mental Health Services Administration, 2021; Thomas et al., 2014). Overall, research has demonstrated the positive impact of MOUD (primarily methadone) on pregnant women by providing a more stable lifestyle and reducing the risks to the mother and child (Tran et al., 2017). Some examples of enhanced neonatal outcomes include improved birth weight and fetal age at birth (Brogly et al., 2014; Burns et al., 2007; Jones et al., 2005).

In this study, we draw from a healthcare disparities conceptual framework that identifies three stages to research disparities: (a) detect health care disparities in a vulnerable population; (b) understand client risk and program capacity factors; and (c) reduce disparities through policy and programmatic interventions (Kilbourne et al., 2006). We use the first two stages of the framework to (1) detect disparities in wait time and retention in OUD treatment, and (2) understand which factors are associated with wait time and retention for pregnant and non-pregnant women with OUD.

We examined disparities in access and retention for pregnant and non-pregnant women in opioid treatment programs in a diverse community sample. We seek to identify factors affecting access and retention for pregnant women with OUD and point to strategies to improve treatment outcomes. Our research questions focus on: (1) What disparities exist in wait time and retention for pregnant women in OUD treatment? and (2) What factors are associated with wait time and retention for pregnant women with OUD?

## 2. Material and methods

### 2.1. Data and sample

We used multi-year client and program data from Los Angeles County Participant Reporting System (LACPRS) and Integrated Substance Abuse Treatment to Eliminate Disparities (iSATed) Program Survey. Data were collected for LACPRS through interviews with intake counselors at admission (Guerrero et al., 2021; Marsh et al., 2021). Information collected included individual characteristics such as demographic characteristics (age, gender, race/ethnicity), education and employment, psychosocial characteristics (e.g., mental illness and family structure), housing status, measures of severity (e.g., age started using primary drug, days using primary drug in last 30 days, number prior treatment episodes), Medi-Cal eligibility, treatment type (whether outpatient MOUD, outpatient counseling or residential), referral source (whether treatment court-mandated), and medications used (whether methadone, buprenorphine or other). There were 9 waves of data collection including consecutive years from 2006 to 2011, and then 2013, 2015, and 2017. The sample consisted of 12,558 clients, with 285 being pregnant and 12,273 being non-pregnant. Of pregnant women, 59.7% were non-Latina White, 30.4% were Latina, 4.6% were Black, and 5.3% were Other. Of non-pregnant women, 57.3% were non-Latina White, 28.8% were Latina, 9.5% were Black, and 4.4% were Other.

### 2.2. Analytic approach

We relied on multilevel negative binomial regression to examine the relationship of access and retention with explanatory variables. Both dependent variables (i.e., access and retention), number of waiting days, and number of days in treatment are counts. The multilevel data structure (i.e. client-program) was accounted for by considering clients in the same program and year as a cluster. Correlation among those clients was incorporated when calculating the standard errors of coefficient estimates. Moreover, an overdisperson parameter was examined to see if negative binomial regression was preferred over Poisson regression. We used R package MASS to run our models that included IRR (i.e., incidence rate ratio) which measured in terms of average number of days.

### 2.3. Dependent variables

The first dependent variable, *access*, refers to the number of days clients spent on the waiting list prior to admission to the treatment program. The item asked, “How many days were you on the waiting list before you were admitted to the treatment program?” The second dependent variable, *retention*, refers to the number of days clients were in treatment from entry to exit. These two outcome measures have been used in several national (Askari et al., 2020; Stahler and Mennis, 2020) and regional studies (Guerrero, 2013, 2014, 2013).

### 2.4. Independent variable

The independent variable, pregnancy at admission, was measured as a dichotomous variable (1 = pregnant; 0 = non-pregnant).

### 2.5. Explanatory variables

We adjusted for covariates with documented association with access and retention, covariates that could potentially confound the relation between pregnancy, access, and retention. We controlled for *year*, i.e., whether data were collected in 2011, 2013, 2015 or 2017. Client demographics included *race*/*ethnicity* (*race* includes individuals who identify as non-Latina White, African American and Other; *ethnicity* includes individuals who identify as Latina; non-Latina White served as reference category), with respondents in the Other race category including the 3.8% of the sample who identified as American Indian, Asian, and Mixed race. Consistent with other research approaches, we coded individuals identified as Latina as a primary category regardless of whether the same client also reported a race category. We coded non-Latina White, African American, and Other when the client identified as any race category and did not identify as Latina. The Other category represents clients who did not identify as non-Latina White, African American, and or Latina.

Clients also reported other demographic and psychosocial characteristics including client *age* (coded as a continuous variable), *education* (years in School), *employment* (unemployed = 0, employed = 1); *homeless* (coded no = 0, yes =1 when intake counselor assessed “Is this person homeless?”); *mental illness* (coded no = 0, yes = 1, when client reported “Have you ever being diagnosed with a mental illness?”); *age started using primary drug* (years); *days using primary drug* (number of days of primary substance use during 30 days prior to admission); *number of children under 18 living at home or not; Medi-Cal* (Medicaid program in California) *insurance eligible* (no = 0, yes = 1); *number of prior SUD treatment episodes* (number of prior episodes in any alcohol or drug treatment/recovery program in which the client participated); *treatment type* (outpatient MOUD, outpatient counseling, residential); *court-mandated referral* (no = 0, yes = 1); and *medications* (none, methadone, buprenorphine, other). These covariates have been used in several national (Askari et al., 2020; Stahler and Mennis, 2020) and regional studies (Guerrero 2013; Guerrero et al., 2014).

## 3. Results

### 3.1. Differences in demographic characteristics between pregnant and non-pregnant women

Table 1 shows a comparative analysis of client characteristics by pregnancy status at admission. Pregnant women spend less time waiting for treatment than non-pregnant women (1.9 days versus 3.6 days) and remain in treatment longer than non-pregnant women (51.9 days versus 33.0 days). A higher proportion of pregnant women identified as non-Latina white (59.7%) and Latina (30.4%) compared with non-pregnant women identifying as non-Latina white (57.3%) and Latina (28.8%). They also were statistically more likely to be younger than non-pregnant women (29.4 years versus 38.2 years, *p* < 0.001), to begin using their reported primary drug at a younger age (19.9 years versus 23.2, *p* < 0.001), and to use the primary drug less frequently in the last 30 days (16.2 days versus 21.0 days, *p* < 0.001). Further, there was a statistically significant association between race and pregnancy status at admission (*p* < 0.05) with those identifying as non-Latina White more likely to be pregnant at admission (59.7%). Pregnant women also were statistically more likely to have children under 18 years of age (1.0 versus 0.7, *p* < 0.001) and more likely to be Medi-Cal eligible compared than non-pregnant women (51.2% versus 28.7%, *p* < 0.001).

Table 1 also indicates a statistically significant association between treatment type and pregnancy (*p* < 0.001). Pregnant women compared with non-pregnant were more likely to be served in both MOUD (38.3% versus 25.7%) and counseling programs (18.4% versus 13.6%), but less likely to be served in residential treatment (43.3% versus 60.6%). Additionally, there was a significant association between medications and pregnant women (*p* < 0.001). Pregnant women compared to non-pregnant women were more likely to receive methadone (43.9% versus 35.6%) or no medication (50.5% versus 41.4%). In this sample, only a small number of non-pregnant women reported receiving buprenorphine (2.7%).

### 3.2. Detecting and understanding factors associated with treatment access and retention for pregnant women

Table 2 shows factors were related to access (days on the waiting list) and retention for pregnant clients using negative binomial models. Older pregnant women and homeless pregnant women were more likely to spend time on the waiting list (IRR = 1.113, *p* < 0.01 and IRR = 3.138, *p* < 0.05, respectively). Pregnant women who started using opioids at a younger age spent less time on the waiting list (IRR = 0.892, *p* < 0.01). Compared with those receiving residential treatment, those receiving methadone (IRR = 0.028, *p* < 0.001) or counseling (IRR = 0.454, *p* < 0.001) were less likely to spend time on the wait list.

Table 2 also shows pregnant women were more likely to be retained in treatment if they identified as Latina (IRR = 1.406, *p* < 0.05), experienced mental health issues (IRR = 1.438, *p* < 0.05), had responsibilities for children under 18 living in the home (IRR = 1.126, *p* < 0.05), had prior treatment episodes (a measure of severity) (IRR = 1.052, *p* < 0.01), received methadone treatment (IRR = 5.455, *p* < 0.001) and counseling treatment (IRR = 2.043, *p* < 0.001) compared to residential treatment. In comparison, pregnant women who used opioids more frequently in the 30 days prior to treatment entry (a measure of severity) were less likely to be retained in treatment (IRR = 0.971, *p* < 0.001).

### 3.3. Detecting and understanding factors associated with treatment access and retention for non-pregnant women

Table 3 indicates factors associated with access (days on waiting list) and retention (days in treatment) for non-pregnant women using negative binomial models. Table 3 shows that factors associated with less access to treatment, i.e., spending more time on the wait list for non-pregnant women included older age (IRR = 1.007, *p* < 0.05), identified as Latina (IRR = 1.874, *p* < 0.001), experienced homelessness (IRR = 1.510, *p* < 0.001), prior treatment episodes (IRR = 1.051, *p* < 0.001), and mandated referral (IRR = 1.552, *p* < 0.001). In comparison, factors associated with greater access to treatment, i.e., spending less time on the wait list, for non-pregnant women were being employed (IRR = 0.618, *p* < 0.001), Medi-Cal eligible (IRR = 0.761, *p* < 0.001), receiving methadone (IRR = 0.064, *p* < 0.001) or counseling (IRR = 0.244, *p* < 0.001) (compared to residential).

Non-pregnant women are retained in treatment longer if they were older (IRR = 1.005, *p* < 0.001), identified as Black (IRR = 1.206, *p* < 0.001), experienced homelessness (IRR = 1.211, *p* < 0.001), were Medi-Cal eligible (IRR = 1.219, *p* < 0.001), received methadone (IRR = 2.904, *p* < 0.001) or counseling (IRR = 2.184, *p* < 0.001), and had a mandated referral (IRR = 1.343, *p* < 0.001). In addition, non-pregnant women were retained at a shorter period of time if they were older when began use of primary drug (IRR = 0.993, *p* < 0.001) and had more days of using primary drug prior to entering treatment (IRR = 0.957, *p* < 0.001). Those receiving methadone (IRR = 2.904, *p* < 0.001), counseling (IRR = 2.184, *p* < 0.001), and had mandated referrals (IRR = 1.343, *p* < 0.001) were likely to stay in treatment longer.

## 4. Discussion

In this paper, we examined differences between pregnant and non-pregnant women in wait time and retention in OUD treatment with the goal of detecting and understanding potential disparities. Three major findings emerged from this analysis. First, pregnant women in OUD treatment have shorter wait times for entering treatment and, once they enter, have longer retention than non-pregnant women in OUD treatment, a finding consistent with the literature (Albrecht et al., 2011; Jancaitis et al., 2020). Also, a significantly greater proportion of pregnant women (51.2%) compared to non-pregnant women (25.7%) were eligible for Medi-Cal (the California version of Medicaid) that provides expedited access to SUD treatment for pregnant women. Within the sample of pregnant women, however, those identifying as Latina wait longer to enter treatment and remain in treatment longer than those identifying as non-Latina White.

Second, pregnant women are more likely to receive MOUD (methadone), the standard of care, compared with non-pregnant women (40% versus 25%) and receipt of methadone is related to reduced wait time and increased retention, similar to previous research on pregnant women receiving treatment for OUD (Brogly et al., 2014; Burns et al., 2007; Jancaitis et al., 2020). It is also important to note that the 38.3% of pregnant women receiving MOUD is substantially lower than the average national rate of approximately 50% (Short et al., 2018). Third, in this sample of pregnant women receiving treatment for OUD, those with greater health and social needs remain in treatment longer. Specifically, greater severity as reflected in prior treatment episodes, mental health issues, and children under 18, are retained longer in treatment.

It is well-known that Medicaid, public health insurance in the U.S., plays an important role in increasing access to SUD treatment (Guerrero, 2013; Andrews et al., 2019). In this sample, a significantly greater proportion of pregnant women (51.2%) compared to non-pregnant women (25.7%) were eligible for Medi-Cal (the California version of Medicaid) which may account for their greater access to treatment (shorter wait times) compared with non-pregnant women. Medicaid eligibility requirements and service provisions are specific to each state and, compared with many states, Medi-Cal eligibility requirements and pregnancy-related services are generous.

Eligible pregnant women in California are required to enroll in a Medi-Cal managed care health plan unless they opt to remain with the physican in a Fee-for-Service plan throughout their pregnancy and postpartum period (DHCS, 2021). Pregnancy-related services are defined as services required to assure the health of the pregnant women and the fetus including prenatal care, services for other conditions that might complicate the pregnancy, labor, delivery, postpartum care, and family planning services. Drug coverage, prescribed for pregnancy-related services and dispensed within the eligibility time frame, includes the full Medi-Cal pharmaceutical benefits. Overall, study findings reinforce the importance of expanding Medicaid in the U.S. in order to improve access to the standard of care for pregnant women in OUD treatment, treatment that includes MOUD along with comprehensive services to assure the health of the pregnant woman and fetus.

Although all pregnant women in OUD treatment have shorter wait times for entering treatment, pregnant women identifying as Latina wait longer to enter treatment and are retained in treatment longer than those identifying as non-Latina White. Numerous studies of gender disparities in access and retention in SUD treatment have pointed to the particular vulnerability of Latina women (Guerrero et al., 2013; Marsh et al., 2004). Latinas received fewer social services but benefited greatly when they did receive them (Marsh et al., 2021). Latinas may have significant need for ancillary services to access and stay in OUD treatment. Programs providing services to these women in low income minority communities may not respond to their service needs, resulting in poor outcomes and unmet treatment goals (Marsh et al., 2021). All of these findings point to the particular vulnerabilities of pregnant women identifying as Latina and the need for the provision of both integrated social services as well as culturally competent services to improve access for all clients (Guerrero et al., 2021).

Findings further reinforce previous research that methadone is associated with longer treatment retention, as retention has been shown to improve pregnancy outcomes (Jancaitis et al., 2020). In this diverse sample from low income, urban communities in Los Angeles County, pregnant women who received outpatient methadone were more likely to have access (i.e., wait fewer days) for treatment and remain in treatment longer. This finding is consistent with research available from the 1970’s indicating methadone is the standard of care for pregnant women (ACOG Committee on Health Care for Underserved Women, & American Society of Addiction Medicine, 2012). Despite this evidence, misconceptions and stereotypes remain that methadone results in substituting one drug for another and may have adverse effects on the infant (Substance Abuse and Mental Health Services Administration, 2021). The finding that pregnant women in this sample are more likely than non-pregnant women to receive MOUD (methadone) (40% versus 25%) may indicate progress is being made in combatting misconceptions and stereotypes in the treatment of pregnant women with OUD. Additionally, these results contribute evidence supporting the use of MOUD (methadone) as an appropriate treatment for increasing access and retention for pregnant women with OUD.

Finally, study findings point to the value of comprehensive services to address the substantial health and social needs of pregnant women with OUD. Numerous studies indicate lack of mental health services, childcare, and parenting support are significant barriers to treatment access and retention for women, especially pregnant and parenting women (Sutter et al., 2017; King et al., 2015). When comprehensive services are available to address these barriers, outcomes improve (Marsh et al., 2004; Marsh et al., 2009; Moreland and McRae-Clark, 2018). It could be that for both pregnant and non-pregnant women with OUD factors such as, lower educational attainment, employment challenges, and mental health issues accentuate the need for comprehensive services (Cao et al., 2011; Guerrero et al., 2014; Marsh et al., 2004). Despite the demonstrated need for treatment programs that provide gender-sensitive services including mental health services, family support, and child care, programs that provide these services are limited and on the decline (Terplan et al., 2015; Brown et al., 2011; Patrick et al., 2019).

This study assessing differences between pregnant and non-pregnant women in opioid treatment programs in Los Angeles County has both strengths and limitations. One major strength of this study is the relatively large and diverse sample of both pregnant and non-pregnant women entering treatment in Los Angeles County between 2006 and 2017 when the opioid epidemic was peaking and when many policy and programmatic changes were being implemented to address it. This sample permitted an analysis of gender disparities that included an analysis of gender and race/ethnic interactions. A second strength derives from the inclusion of both pregnant and non-pregnant women in opioid treatment in our sample. This permitted comparison of wait time and retention and factors that affected pregnant and non-pregnant women. It also enabled us to understand the distinct barriers and facilitators to treatment for both pregnant and non-pregnant women in treatment. Notably, findings on barriers and facilitators of this regional sample of non-pregnant women were consistent with findings from national studies of women in substance use treatment (Guerrero et al., 2014; Marsh et al., 2004, 2009).

Limitations of this study also require consideration when interpreting the results. First, the results are not representative or generalizable to all pregnant and non-pregnant women in substance use treatment across the nation, as the sample was localized to OUD treatment programs in Los Angeles, County. A second limitation is related to the lack of analysis of change over time when resources and approaches used to address the opioid epidemic were increasing and changing. Lastly, the LACPRS data is administrative data that relies on self-report questionnaires, requiring the need for caution with interpretation due to validity and reliability.

## 5. Conclusions

The results of this study support the need for specific strategies for improving access and retention to OUD treatment for pregnant women. These strategies include continued advocacy for the standard of care for pregnant women with OUD that includes medication along with comprehensive services to address health, mental health and social needs. The importance of Medi-Cal for improving access and retention for both pregnant and non-pregnant women –but especially pregnant women–with OUD in this sample speaks to the need for continued expansion of Medicaid programs across the U.S. Overall, these findings suggest the need to analyze change over time in disparities in treatment access and retention as well as to design outpatient OUD programs for both pregnant women and non-pregnant women with OUD that are comprehensive, gender-sensitive and evidence-based.

## Figures and Tables

**Table 1:** Comparative analysis of client characteristics by pregnant status.

	Pregnant at admission (*N* = 285)	Not pregnant at admission (*N* = 12,273)
Days in wait**	1.9 (9.2)	3.6 (10.8)
Treatment duration***	51.9 (68.8)	33.0 (53.6)
Year***	5.6 (2.8)	4.7 (2.5)
Age***	29.4 (6.6)	38.2 (11.9)
Race*		
White	169 (59.7%)	6888 (57.3%)
Black	13 (4.6%)	1146 (9.5%)
Latina	86 (30.4%)	3459 (28.8%)
Other	15 (5.3%)	525 (4.4%)
Education (years)	11.7 (2.4)	11.9 (2.4)
Employed	24 (8.4%)	893 (7.4%)
Homeless	74 (26%)	3039 (25.2%)
Mental health issues	85 (29.8%)	4160 (34.5%)
Age using primary drug**	19.9 (5.9)	23.2 (9.5)
Days using primary drug***	16.2 (13.5)	21.0 (12.4)
# Children under 18***	1.0 (1.5)	0.7 (1.8)
Medi-cal eligible***	146 (51.2%)	3458 (28.7%)
# prior episodes	3.0 (4.1)	3.1 (4.7)
Treatment type***		
MOUD	106 (38.3%)	3047 (25.7%)
counseling	51 (18.4%)	1614 (13.6%)
Residential	120 (43.3%)	7182 (60.6%)
Mandated referral	29 (10.2%)	1171 (9.7%)
Medications***		
None	144 (50.5%)	5001 (41.4%)
Methadone	125 (43.9%)	4299 (35.6%)
Buprenorphine	0 (0%)	319 (2.7%)
Other	16 (5.6%)	2448 (20.3%)

* *p* < 0.05,.

** *p* < 0.01,.

*** *p* < 0.001.

**Table 2:** Negative binomial models for wait time and retention within pregnant women at admission.

	Wait time		Retention	
	IRR	95% CI	IRR	95% CI
Year	0.883	0.724, 1.077	0.973	0.913, 1.036
Age	1.113**	1.027, 1.206	0.991	0.967, 1.017
Race^a^				
Black	0.162	0.006, 4.257	0.840	0.362, 1.950
Latina	2.731	0.926, 8.059	1.406*	1.000, 1.977
Other	6.363	0.917, 44.156	0.745	0.349, 1.589
Education (years)	1.031	0.843, 1.261	1.047	0.979, 1.119
Employed	1.263	0.199, 8.018	1.716	0.958, 3.073
Homeless	3.138*	1.068, 9.222	1.167	0.823, 1.657
Mental health issues	0.406	0.139, 1.183	1.438*	1.035, 1.997
Age using primary drug	0.892**	0.818, 0.973	1.007	0.979, 1.036
Days using primary drug	1.012	0.970, 1.055	0.971***	0.958, 0.984
# Children under 18	1.156	0.857, 1.560	1.126*	1.018, 1.245
Medi-cal eligible	2.314	0.713, 7.509	0.882	0.599, 1.298
# prior episodes	0.898	0.798, 1.011	1.052**	1.013, 1.093
Treatment type^b^				
MOUD	0.028***	0.006, 0.127	5.455***	3.464, 8.590
counseling	0.454***	0.382, 0.541	2.043***	1.890, 2.207
Mandated referral	2.370	0.540, 10.403	0.735	0.448, 1.203
# observations	*N* = 275		*N* = 232	

a White as reference;.

b Residential as reference.

* *p* < 0.05,.

** *p* < 0.01,.

*** *p* < 0.001.

**Table 3:** Negative binomial models for wait time and retention within non-pregnant women at admission.

	Wait time		Retention	
	IRR	95% CI	IRR	95% CI
Year	0.862***	0.844, 0.881	1.000	0.992, 1.009
Age	1.007*	1.001, 1.012	1.005***	1.003, 1.007
Race^a^				
Black	1.039	0.854, 1.263	1.206***	1.121, 1.299
Latina	1.874***	1.657, 2.119	0.982	0.936, 1.030
Other	1.196	0.923, 1.548	1.035	0.936, 1.145
Education (years)	0.995	0.973, 1.018	0.992	0.983, 1.001
Employed	0.618***	0.500, 0.765	0.871***	0.804, 0.943
Homeless	1.510***	1.339, 1.704	1.211***	1.154, 1.271
Mental health issues	0.959	0.858, 1.072	0.973	0.932, 1.017
Age using primary drug	1.005	0.999, 1.011	0.993***	0.991, 0.996
Days using primary drug	1.000	0.995, 1.005	0.957***	0.955, 0.959
# Children under 18	1.002	0.975, 1.030	0.975***	0.964, 0.986
Medi-cal eligible	0.761***	0.664, 0.872	1.219***	1.158, 1.283
# prior episodes	1.051***	1.040, 1.062	1.004	1.000, 1.009
Treatment type^b^				
MOUD	0.064***	0.054, 0.075	2.904**	2.742, 3.075
counseling	0.244***	0.203, 0.292	2.184***	2.034, 2.344
Mandated referral	1.552***	1.286, 1.873	1.343***	1.245, 1.448
# observations	*N* = 11,792		*N* = 11,594	

a White as reference;.

b Residential as reference.

* *p* < 0.05,.

** *p* < 0.01,.

*** *p* < 0.001.

## References

[R1] ACOG Committee on Health Care for Underserved Women, & American Society of Addiction Medicine, 2012. ACOG Committee Opinion No. 524: opioid abuse, dependence, and addiction in pregnancy. Obstet. Gynecol 119, 1070–1076. doi: 10.1097/AOG.0b013e318256496e. 22525931

[R2] Agency for Healthcare Research and Quality, Healthcare Cost and Utilization Project (HCUP), 2018. HCUP-US Databases. Agency for Healthcare Research and Quality, Rockville, MD Retrieved from https://www.hcup-us.ahrq.gov/databases.

[R3] Albrecht J, Lindsay B, Terplan M, 2011. Effect of waiting time on substance abuse treatment completion in pregnant women. J. Subst Abuse Treat 41, 71–77. doi:10.1016/j.jsat.2011.01.015. 21353440

[R4] Andrews CM, Pollack HA, Abraham AJ, Grogan CM, Bersamira CS, D’Aunno T, Friedmann PD, 2019. Medicaid coverage in substance use disorder treatment after the affordable care act. J. Subst Abuse Treat 102, 1–7. doi: 10.1016/j.jsat.2019.04.002. 31202283 PMC6578872

[R5] Askari MS, Martins SS, Mauro PM, 2020. Medication for opioid use disorder treatment and specialty outpatient substance use treatment outcomes: differences in retention and completion among opioid-related discharges in 2016. J. Subst Abuse Treat 114, 108028. 32527510 10.1016/j.jsat.2020.108028 PMC7328306

[R6] Barbosa-Leiker C, Campbell ANC, McHugh RK, Guille C, Greenfield SF, 2020. Opioid use disorder in women and the implications for treatment. PRCP 3, 3–11. 34870109 10.1176/appi.prcp.20190051 PMC8639162

[R7] Brogly SB, Saia KA, Walley AY, Du HM, Sebastiani P, 2014. Prenatal buprenorphine versus methadone exposure and neonatal outcomes: systematic review and meta-analysis. Am. J. Epidemiol 180, 673–686. doi:10.1093/aje/kwu190. 25150272

[R8] Brown JD, Vartivarian S, Alderks CE, 2011. Child care in outpatient treatment facilities for women: findings from the 2008 national survey of substance abuse treatment services. J. Behav. Health Serv. Res 38, 478–487. 21293975 10.1007/s11414-011-9235-1

[R9] Burns L, Mattick RP, Lim K, Wallace C, 2007. Methadone in pregnancy: treatment retention and neonatal outcomes. Addiction 102, 264–270. doi:10.1111/j.1360-0443.2006.01651.x. 17222281

[R10] Cao D, Marsh JC, Shin H−C, Andrews C, 2011. Improving health and social outcomes with targeted services in comprehensive substance abuse treatment. Am Drug Alcohol Abuse 37, 250–258. doi:10.3109/00952990.2011.591016. 21699362

[R11] California Department of Health Care Services (DHCS). 2021. Full scope Medi-cal Coverage and affordability and benefit program for low- income pregnant women and newly qualified immigrants. Retrieved from: https://www.dhcs.ca.gov/services/medical/Pages/Affordability-and-Benefit-Program.aspx.

[R12] Centers for Disease Control and Prevention. 2020a. Overdose deaths accelerating during COVID-19. Retrieved from: https://www.cdc.gov/media/releases/2020/p1218-overdose-deaths-covid-19.html

[R13] Centers for Disease Control and Prevention. 2020b. Pregnancy and opioid use. Retrieved from: https://www.cdc.gov/pregnancy/opioids/data.html

[R14] Guerrero EG, 2013. Enhancing access and retention in substance abuse treatment: the role of Medicaid payment acceptance and cultural competence. Drug Alcohol Depend. 132, 555–561. doi:10.1016/j.drugalcdep.2013.04.005. 23669458

[R15] Guerrero EG, Marsh JC, Duan L, Oh C, Perron B, Lee B, 2013. Disparities in completion of substance abuse treatment between and within racial and ethnic groups. Adm Policy Ment. Health 48, 1450–1467. doi:10.1111/1475-6773.12031. PMC3725534 23350871

[R16] Guerrero EG, Marsh JC, Cao D, Shin HC, Andrews C, 2014. Gender disparities in utilization and outcome of comprehensive substance abuse treatment among racial/ethnic groups. J. Subst. Abus. Treat 46, 584–591. 10.1016/j.jsat.2013.12.008 PMC3972361 24560127

[R17] Guerrero EG, Amaro HA, Kong Y, Khachikian T, Marsh JC, 2021. Gender disparities in opioid treatment progress in methadone versus counseling. Subst. Abuse Treat PR 16. doi:10.1186/s13011-021-00389-4. PMC8220800 34162420

[R18] Haight SC, Ko JY, Tong VT, Bohm MK, Callaghan WM, 2018. Opioid use disorder documented at delivery hospitalization–United States, 1999–2014. MMWR Morb. Mortal. Wkly. Rep 67, 845–849. doi:10.15585/mmwr.mm6731a1externalicon. 30091969 PMC6089335

[R19] Harter K, 2019. Opioid use disorder in pregnancy. MHC 9, 359–372. doi: 10.9740/mhc.2019.11.359. 31857932 PMC6881108

[R20] Jancaitis B, Kelpin S, Masho S, May J, Haug NA, Svikis D, 2020. Factors associated with treatment retention in pregnant women with opioid use disorders prescribed methadone or electing non-pharmacological treatment. Women Health 60, 1–11. doi:10.1080/03630242.2019.1610829. 31068095 PMC6842074

[R21] Jones HE, Johnson RE, Jasinski R, O’Grady KE, Chisholm CA, Choo RE, Milio L, 2005. Buprenorphine versus methadone in the treatment of pregnant opioid- dependent patients: effects on the neonatal abstinence syndrome. Drug Alcohol Depend. 79, 1–10. 15943939 10.1016/j.drugalcdep.2004.11.013

[R22] Kilboume AM, Switzer G, Hyman K, Crowley-Matoka M, Fine MJ, 2006. Advancing health disparities research in the health care system: a conceptual framework. Am. J. Public Health 96, 2113–2121. 17077411 10.2105/AJPH.2005.077628 PMC1698151

[R23] King PAL, Duan L, Amaro H, 2015. Clinical needs of in-treatment pregnant women with co-occurring disorders: implications for primary care. Matern. Child Health J 19, 180–187. doi:10.1007/s10995-014-1508-x, Doi:. 24770992

[R24] Klaman SL, Isaacs K, Leopold A, Perpich J, Hayashi S, Vender J, Campopiano M, Jones HE, 2017. Treating women who are pregnant and parenting for opioid use disorder and the concurrent care of their infants and children: literature review to support national guidance. J. Addict. Med 11, 178–190. doi: 10.1097/ADM.0000000000000308. 28406856 PMC5457836

[R25] Marsh JC, Amaro H, Kong Y, Khachikian T, Guerrero E, 2021. Gender disparities in access and retention in outpatient methadone treatment for opioid use disorder in low-income urban communities. J. Subst. Abus. Treat 127,108399. 10.1016/j.jsat.2021.108399 PMC8994515 34134873

[R26] Marsh JC, Cao D, D’Aunno T, 2004. Gender differences in the impact of comprehensive services in substance abuse treatment. J. Subst. Abus. Treat 27, 289–300. doi:10.1016/j.jsat.2004.08.004. 15610830

[R27] Marsh JC, Cao D, Shin HC, 2009. Closing the need—Service gap: gender differences in matching services to client needs in comprehensive substance abuse treatment. Soc. Work Res 33, 183–192. 21566721 10.1093/swr/33.3.183 PMC3091618

[R28] Marsh JC, D’Aunno TA, Smith BD, 2000. Increasing access and providing social services to improve drug abuse treatment for women with children. Addiction 95, 1237–1247. doi:10.1046/j.1360-0443.2000.958123710.x. 11092071

[R29] Moreland AD, McRae-Clark A, 2018. Parenting outcomes of parenting interventions in integrated substance-use treatment programs: a systematic review. J. Subst. Abus. Treat 89, 52–59. 10.1016/j.jsat.2018.03.005 PMC5939592 29706173

[R30] Patrick SW, Buntin MB, Martin PR, Scott TA, Dupont W, Richards M, Cooper WO, 2019. Barriers to accessing treatment for pregnant women with opioid use disorder in Appalachian states. Subst Abus. 40, 356–362. 29949454 10.1080/08897077.2018.1488336 PMC9069995

[R31] Short VL, Hand DJ, MacAfee L, Abatemarco DJ, Terplan M, 2018. Trends and disparities in receipt of pharmacotherapy among pregnant women in publically funded treatment programs for opioid use disorder in the United States. J. Subst. Abus. Treat 89, 67–74. doi:10.1016/j.jsat.2018.04.003. 29706175

[R32] Stahler GJ, Mennis J, 2020. The effect of medications for opioid use disorder (MOUD) on residential treatment completion and retention in the US. Drug Alcohol Depend. 212, 108067. 32445926 10.1016/j.drugalcdep.2020.108067

[R33] Substance Abuse and Mental Health Services Administration. 2021. Buprenorphine. Retrieved from: https://www.samhsa.gov/medication-assisted-treatment/medications-counseling-related-conditions/buprenorphine

[R34] Sutter MB, Gopman S, Leeman L, 2017. Patient-centered care to address barriers for pregnant women with opioid dependence. Clin. Obstet. Gynecol 44, 95–107. 10.1016/j.ogc.2016.11.004 28160896

[R35] Terplan M, Longinaker N, Appel L, 2015. Women-centered drug treatment services and need in the United States, 2002–2009. Am. J. Public Health 105, e50–e54. 26378825 10.2105/AJPH.2015.302821 PMC4605167

[R36] The American College of Obstetricians and Gynecologists. 2017. Opioid use and opioid use disorder in pregnancy. Retrieved from: https://www.acog.org/clinical/clinical-guidance/committee-opinion/articles/2017/08/opioid-use-and-opioid-use-disorder-in-pregnancy?utm_source=redirect&utm_medium=web&utm_campaign=otn

[R37] Thomas CP, Fullerton CA, Kim M, Montejano L, Lyman DR, Dougherty RH, Daniels AS, Ghose SS, Delphin-Rittmon ME, 2014. Medication-assisted treatment with buprenorphine: assessing the evidence. Psychiatr. Serv 65, 158–170. doi: 10.1176/appi.ps.201300256. 24247147

[R38] Tran TH, Griffin BL, Stone RH, Vest KM, Todd TJ, 2017. Methadone, buprenorphine, and naltrexone for the treatment of opioid use disorder in pregnant women. Pharmacotherapy 37, 824–839. doi:10.1002/phar.1958. 28543191

